# Cold SiO_2_-rich slabs reaching the CMB revealed by the seifertite phase boundary

**DOI:** 10.1038/s41598-026-54731-6

**Published:** 2026-05-30

**Authors:** Ryosuke Sinmyo, Saori Kawaguchi-Imada, Rei Sato, Keisuke Otsuru, Kenji Kawai, Hiroshi Sakuma, Shigeru Suehara, Takayuki Ishii, Shuhou Maitani

**Affiliations:** 1 Department of Physics, School of Science and Technology, Meiji University, 1-1-1 Higashi Mita, Tama-ku, Kawasaki, Kanagawa 214-8571 Japan; 2 Japan Synchrotron Radiation Research Institute, 1-1-1 Kouto, Sayo-cho, Sayo-gun, Hyogo, 679-5198 Japan; 3 Office of Institutional Advancement and Communications Unit for Research and Development of Hydrogen Energy Materials and Next Generation Battery, Kyoto University, Hyogo, Japan; 4 Department of Earth and Planetary Science, School of Science, The University of Tokyo, 7-3-1 Hongo, Bunkyo-ku, Tokyo, 113-0033 Japan; 5 Environmental Circulation Composite Materials Group, National Institute for Materials Science, Tsukuba, Ibaraki 305-0044 Japan; 6 Institute for Planetary Materials, Okayama University, Misasa, Tottori 682-0193 Japan; 7 Present Address: Department of Earth and Planetary Sciences, Faculty of Science, Hokkaido University, Sapporo, Hokkaido 060-0810 Japan

**Keywords:** Physics, Solid Earth sciences

## Abstract

A cold silica (SiO_2_)-rich subducted slab creates notable heterogeneity above the core-mantle boundary (CMB), influencing the evolution of the Earth’s mantle. This slab may exhibit characteristic anomalies in the longitudinal and shear wave seismic velocity above the CMB, attributed to the SiO_2_ phase transition into its dense polymorph, seifertite. However, the transition depth remains unclear due to the often-observed metastable phases in experiments. To address this long-standing challenge, we conducted laser-heated diamond anvil cell experiments with synchronised rapid X-ray diffraction measurements alongside theoretical calculations. The slope of the seifertite phase boundary was less steep than previously estimated, and consequently, the temperature profile of the slab crosses the boundary twice, like the post-perovskite transition. We observed a decrease in shear wave velocity beneath Hawaii and Central America, and we found anti-correlation in seismic wave velocities matching the depth range of the seifertite transition in a cold slab beneath Central America. This may provide evidence that a cold SiO_2_-rich slab descends towards the CMB.

**Supplementary Information:**

The online version contains supplementary material available at 10.1038/s41598-026-54731-6.

## Introduction

Subduction is a distinctive geological phenomenon of the Earth among the planets. Global seismic tomography studies have suggested that subducted slabs likely reach down to the bottom of the mantle over geological timescales^1–3^. The fate of the iron- and silica (SiO_2_)-rich subducted mid-oceanic ridge basalt (MORB) strongly affects the physical evolution of the Earth’s mantle, as these materials have anomalous thermal conductivity^4^ and viscosity^5^, dominating mantle convection and core dynamo of the Earth^6^. However, the descent of the subducted MORB to the core-mantle boundary (CMB) is not directly proven so far for two main reasons: (1) the detailed seismic observations are limited to shear (S)-wave velocity, and (2) the depth of a key phase transition in SiO_2_ remains unclear in experimental studies, although it is expected to occur in the lowermost mantle. In most previous seismic observations, the structure of the lower mantle has been explored based on the S-wave velocity, which is sensitive to thermal/chemical heterogeneity and mineral phase transitions. Mineral physics studies show that low-temperature and iron-poor materials induce fast S-wave velocity anomalies, and the bridgmanite to post-perovskite phase transition leads to a positive (fast) discontinuity in the wave velocity several hundred kilometres above the CMB^7–10^. Yet the post-perovskite transition induces a less significant discontinuity in the longitudinal (P)-wave than the S-wave. The double-crossing of the post-perovskite phase boundary provides a strong framework for understanding the thermal and chemical heterogeneity above the CMB^7,9,11^. Nevertheless, conventional seismic observations have intrinsic difficulty distinguishing chemical/thermal anomalies solely based on S-wave velocity. Most studies have inferred P-wave velocity models with less spatial resolution than S-velocity models because P-wave data are generally not sensitive to the region immediately above the CMB. However, P-wave velocity has the potential of distinguishing chemical and thermal heterogeneity when it is combined with the S-wave velocity. This combination could allow for direct proof of the descent of the subducted MORB toward the CMB through observations. On the other hand, subducted MORB contains more than 20% SiO_2_ phase^12,13^, which undergoes a phase transition from a mineral with relatively high symmetry (stishovite) to a distorted phase (CaCl_2_-type phase) at the mid-depths of the lower mantle^14^. The CaCl_2_-type phase further transforms into a denser mineral with an α-PbO_2_-type crystal structure (seifertite) under high-pressure conditions corresponding to the bottom of the mantle^15,16^. The seifertite transition shows a unique feature in terms of the wave velocity above the CMB: a fast anomaly in the P-wave and a slow anomaly in the S-wave^16,17^. This phenomenon contrasts with the post-perovskite transition, which is characterised by fast anomalies in both the P- and S-waves^18^. Although the anti-correlation of wave velocities in the seifertite transition can be considered key to exploring the fate of the subducted slab, the pressure and temperature conditions of the seifertite transition are still highly controversial in both experimental and theoretical studies^19–23^. This controversy likely arises because of the similar total free energy and/or the kinetic barrier of the high-pressure phases in the SiO_2_ system. Various metastable phases in SiO_2_ system at high pressures inhibit the precise determination of the seifertite phase boundary^24–26^. Some previous studies have shown the coexistence of seifertite and CaCl_2_-type phases under single pressure and temperature conditions despite the fact that this phenomenon should be forbidden according to the Gibbs phase rule^19,22^, likely due to the metastable phase in SiO_2_ and undesired thermal gradients and instability during prolonged laser heating. In this work, we determined the seifertite boundary by state-of-the-art experiments, alongside theoretical calculations. We determined the precise phase boundary with a minimised effect of the metastable phase. Then, we explored the fate of the subducted slab through a combination of experimental results and seismic observations. We found an anti-correlation in the P- and S-wave anomalies beneath Central America at depth, which was consistent with the experimentally determined seifertite transition in the cold slab. This observation provides the first direct evidence of a subducting SiO_2_-rich cold slab above the CMB.

## Results

### Determination of the seifertite boundary

**Fig. 1:**
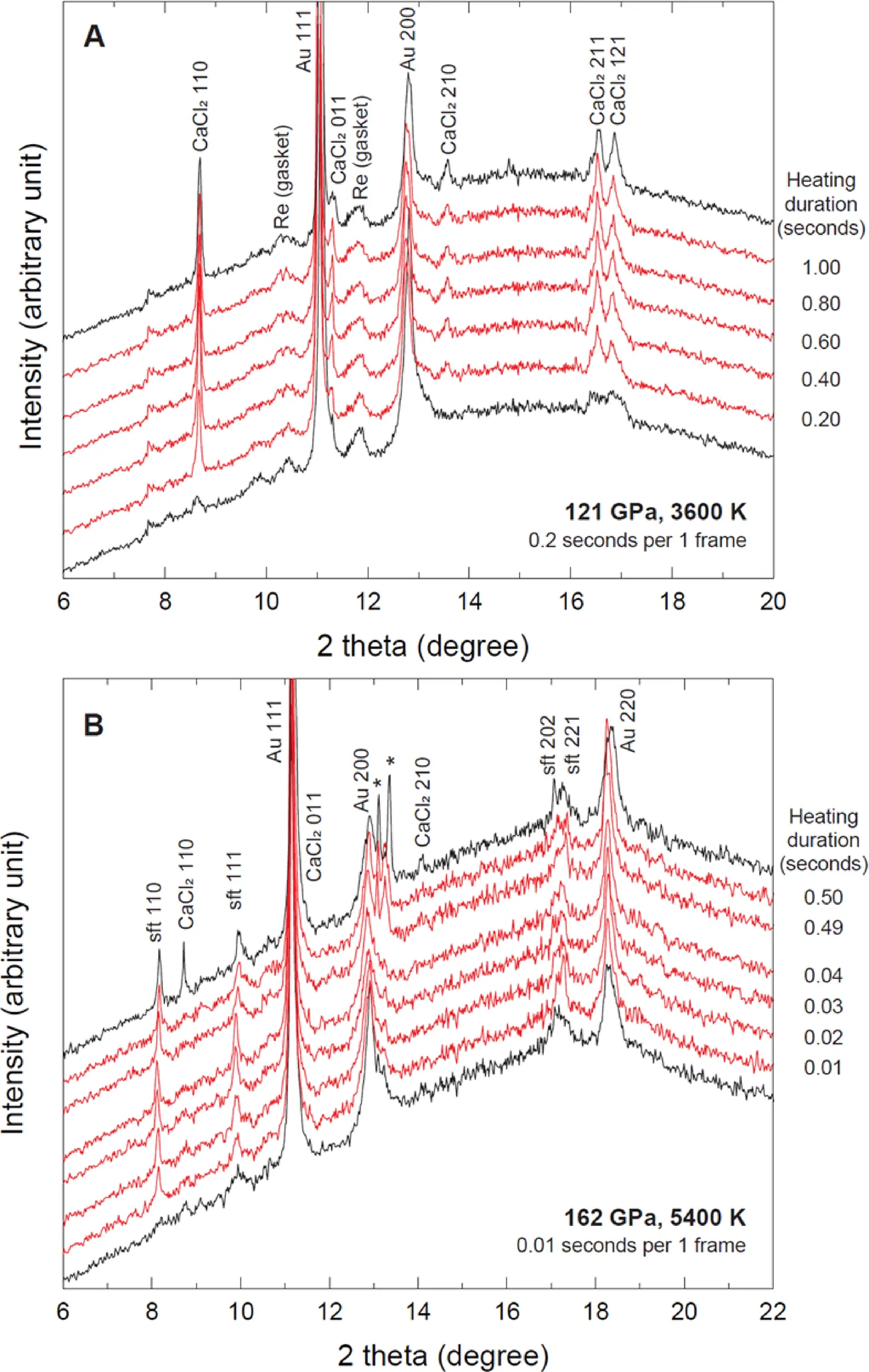
X-ray diffraction measurements. XRD patterns of SiO_2_ at high pressures and temperatures obtained via laser-heated diamond anvil cell experiments. The red lines are the patterns taken during heating, and the black lines are taken at 300 K. (**A**) Run at 121 GPa and 3600 K. The XRD pattern taken before heating shows the weak peaks of the CaCl_2_-type phase and seifertite after cold compression. High-temperature XRD patterns revealed that the CaCl_2_-type phase grew after the onset of heating and reached a maximum abundance 400 milliseconds after the onset of heating. (**B**) Run at 162 GPa and 5400 K. Weak peaks of the CaCl_2_-type phase and seifertite were observed after cold compression. Seifertite grew at high temperatures and reached a maximum abundance 30 milliseconds after the onset of heating. Two new peaks (marked by stars) appeared after approximately 400 milliseconds, which were likely caused by the reaction of the gasket material or diamond anvil. The metastable CaCl_2_-type phase abruptly grew just after the temperature was quenched. In all the experimental runs, the stable phases were determined from the XRD pattern taken at the very beginning of the heating.

**Fig. 2:**
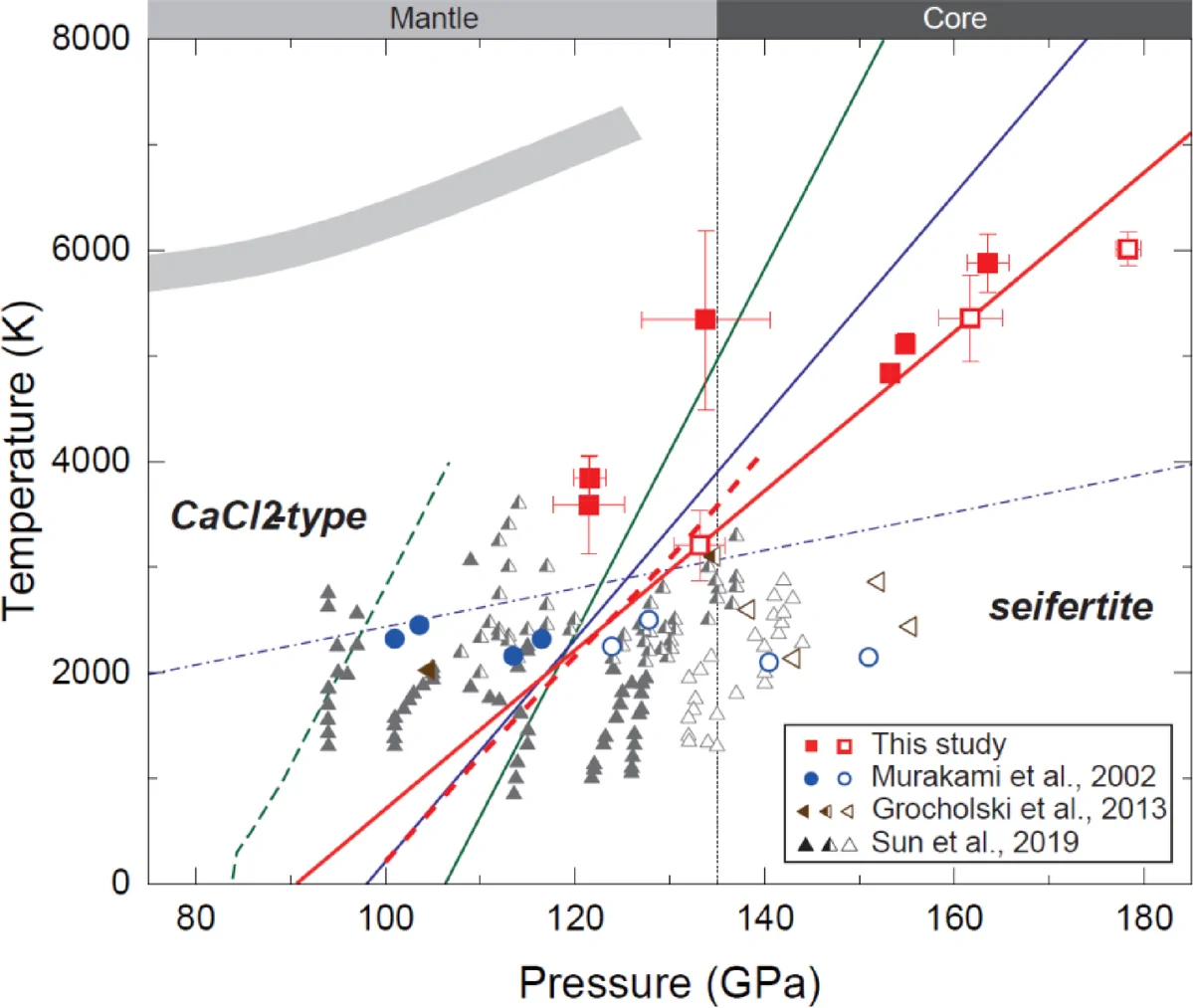
Phase diagram of SiO_2_ around the CMB. The red symbols represent the experimental results of this study. Bold red line (experiments) and broken red line (theoretical calculations) are the boundary between CaCl_2_-type phase and seifertite by this study. The blue^20^, brown^19^, and grey^22^ symbols represent previous experimental studies. The open symbols represent seifertite and the filled symbols represent the CaCl_2_-type phase. The half-filled symbols show the experimental conditions where the seifertite and CaCl_2_-type phases were observed. The green broken^21^ and solid^23^ lines are the previous studies by theoretical calculations. The blue solid thick line is the seifertite boundary^20^, and the blue chain line is the extrapolated high-temperature limit of the synthesis condition of the metastable seifertite^20^. The grey thick line is the proposed melting curves of SiO_2_^79^. The thin dotted line is the pressure condition of the CMB.

We conducted nine separate laser-heated diamond anvil cell experiments employing a rapid synchronised X-ray diffraction measurement technique to minimise the kinetic problem that arises when determining the phase boundary of SiO_2_ at the CMB conditions. A pre-synthesised single-phase seifertite was used as the starting material to avoid encountering the undesired metastable crystal structure developed in the SiO_2_ system under compression^24,26^. Under compression at 300 K, seifertite remained in the original crystal structure, whereas the diffraction peaks were generally weak. We observed very weak peaks of the CaCl_2_-type phase in some experimental runs in addition to seifertite under compression around the stability field of the CaCl_2_-type phase (Fig. 1). In contrast, the conventional starting materials used in previous studies, such as α-quartz, tridymite, cristobalite, and glass, often form metastable phases under cold compression^26,27^. For example, 3 × 2 zig-zag chains of SiO_6_ polyhedra were formed in the phase with *P2*_*1*_*/c* symmetry under the cold compression of α-quartz^26^. The complex combination of the polyhedra in silica results in various metastable phases under high-pressure conditions^24^. Moreover, the SiO_2_ glass exhibits multiple distinct amorphous states (polyamorphism), which are believed to undergo transition under high-pressure conditions^27^. Since we used seifertite as a starting material, the kinetic barrier unrelated to the seifertite transition should be absent in this study. After compression to the desired pressure, the phase relationship of SiO_2_ was determined by using in-situ rapid synchronised X-ray diffraction (XRD) measurements at high pressures and temperatures reaching 178 GPa and 6000 K. We extracted the XRD pattern from the onset of heating to determine the primary stable phase under the investigated pressure and temperature conditions. The measurements were as short as 10 milliseconds and perfectly synchronised with the onset of laser heating. We precisely synchronised all the starting triggers for laser heating, XRD measurement, and temperature determination via the spectroradiometric method. While the heating duration was 0.5–1 s in total, we captured an XRD pattern and a temperature profile just after the onset of heating with an exposure time of 0.01–0.2 s. The undesired effects of temperature instability and chemical heterogeneity in laser-heated diamond anvil cell experiments^28^ were avoided in the results by gating the measurement time to the beginning of heating. We observed that only one phase crystallised and reached its maximum abundance at the early stage of heating (Fig. 1). At approximately 121 GPa, we obtained the XRD pattern and temperature from 0 to 0.2 s after the onset of heating and observed that the single CaCl_2_-type phase was crystallised at 121 GPa and 3600 K (Fig. 1A). Afterwards, we observed the subsequent growth of the CaCl_2_-type phase. The peak intensity reached a maximum value 0.4 s after the onset of heating. No other peaks were observed while the sample was quenched in this run. Only the first measurements were used to determine the seifertite transition boundary. In contrast, a single phase of seifertite grew at 0.01 s after the onset of heating at 162 GPa and 5400 K (Fig. 1B). The peak intensity reached its maximum value at 0.03 s after the onset of heating. Approximately 0.4 s after the seifertite reached its maximum abundance, two new peaks appeared that were likely caused by the reaction of the gasket or anvil material. Moreover, we observed abrupt growth of the CaCl_2_-type phase just after temperature quenching after heating for 0.5 s, whereas this growth was absent during heating. It was considered unreasonable to assume that the CaCl_2_-type phase grew by passing through the stability field during quenching, as it is a higher temperature phase than seifertite. Consequently, the CaCl_2_-type phase was metastable, and the first-grown seifertite was stable at 162 GPa and 5400 K. Our current observations explain why the thermodynamically forbidden two-phase region was observed in previous experimental studies (Fig. 2)^19–22^. The metastable phase could be grown during quenching or fluctuation in temperature during the prolonged laser heating (typically more than several minutes) in the previous conventional laser-heating experimental studies. The growth of the metastable phase led to the misleading observation of a two-phase region as shown in previous experimental studies (Fig. 2). Our rapid measurements minimised these undesired effects during heating. We determined the seifertite phase boundary on the basis of the XRD pattern and temperature taken from the beginning of heating to observe the first crystallised phase (Fig. 2).

### The energetic comparison of the SiO_2_ phases

In addition to the high-pressure experiments, we theoretically investigated the energetic differences between CaCl_2_-type SiO_2_ and seifertite by ab initio and molecular dynamics simulations to address the possible crystallisation of the metastable phases. Density functional theory (DFT) calculations were conducted to obtain the ground-state lattice energy for various unit cell volumes via variable-cell relaxations. Helmholtz free energies were then calculated using a quasi-harmonic approximation with phonon calculations on the basis of density-functional perturbation theory for the optimised structures. The obtained Helmholtz free energies of the seifertite and CaCl_2_-type phases were fitted to the isothermal Vinet equation of state to consider the pressure and volume effects on the free energy (Fig. S1). The phase boundary was determined by the Gibbs free energies of the two phases, and the seifertite boundary was highly consistent with the current experimental results (Figs. S2 and S3). We observed that the difference in the free energy was very small between the two phases under a wide pressure and temperature range around the CMB (Fig. S2). Furthermore, we calculated the intrinsic thermal fluctuations in the SiO_2_ system via Born-Oppenheimer molecular dynamics simulations (Fig. S4). The calculation results revealed that the total energy differences between the two phases were smaller than the kinetic energy intrinsically caused by thermal fluctuations in the microscopic region that was smaller than the ~ 8*8*8 nm^3^ system. In other words, the metastable phase (seifertite, in this case) can crystallise at local ~ 500 nm^3^ scales during the experiment at 135 GPa and 4000 K. Although XRD measurements could not observe such small crystals, the crystal could act as nuclei for subsequent crystal growth when undesired temperature instability or quenching occurs during laser heating. Indeed, the metastable phase was not observed by XRD measurements in this study during heating, and it appeared at the quenching temperature (Fig. 1B). This hypothesis could explain the occurrence of seifertite in natural meteorites and lunar regoliths, which are likely heated and quenched on a short time scale during shock events^29,30^. Since metastable seifertite is crystallised at relatively low pressure^31^, the difference in the free energy should be small between low-pressure phases and seifertite. During a shock event, seifertite can be locally crystallised at high temperatures, and the tiny seifertite acts as a nucleus for subsequent crustal growth during the temperature quench.

### Double crossing of the boundary and the seismic detectability

**Fig. 3:**
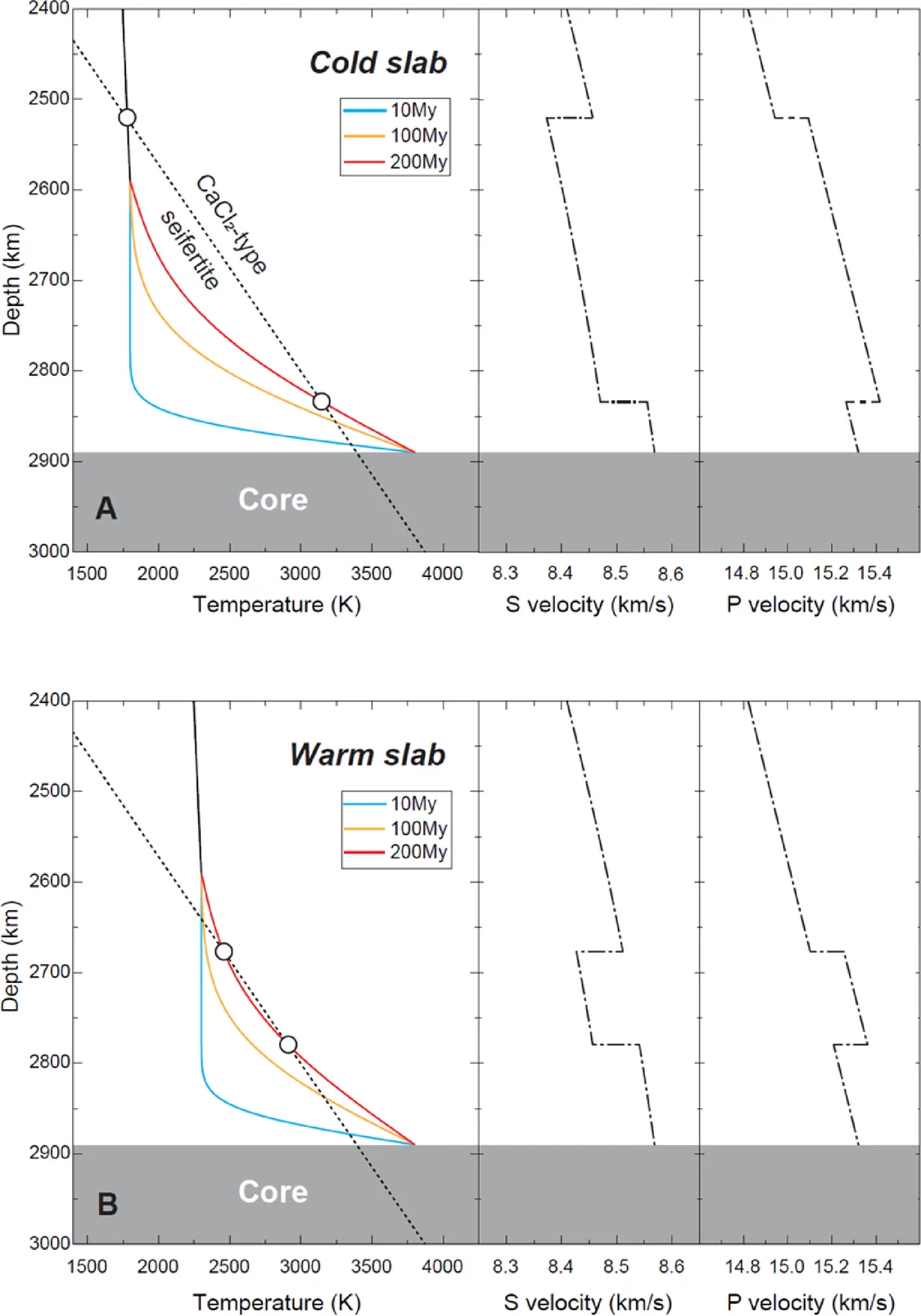
The seifertite boundary and slab temperature. The seifertite boundary and the temperature profiles of the (**A**) cold and (**B**) warm slab above the CMB. The dotted black line is the phase boundary between the CaCl_2_-type phase and seifertite. The solid lines represent the temperature profile estimated by assuming a 300 km thick thermal boundary layer and heat conduction from the core with T_CMB_ = 3900 K^7,35^. Blue, 10 My; yellow, 100 My; red, 200 My. The chain lines are the maximum extent of the S- and P-wave velocity anomalies induced by the SiO_2_ phase transition to seifertite^16^.

The Clapeyron slope of the seifertite transition (13.3(9) MPa/K) was similar to that of the CaCl_2_-type transition from stishovite^32^ and the post-perovskite transition in MgSiO_3_^33^ (Fig. S5). The slope was less steep than the value that was previously estimated^19–23^, and as a result, the temperature of the descending slab likely crosses the obtained boundary twice at approximately 100 km and several hundred kilometres above the CMB (Fig. 3), as in the case of the double-crossing of the post-perovskite transition boundary^11^. We estimated the slab temperature on the basis of the adiabatic geotherm and the thermal conduction from the core through the 300 km thermal boundary above the CMB (Fig. 3). We assumed that there was no external heat source, such as radiogenic heat or lateral heat conduction from the surrounding mantle. The temperatures of the slabs were assumed to be 400 and 900 K colder at the top of the thermal boundary layer than the adiabatic geotherm in the warm and cold slabs, respectively (Fig. 3)^34^. The temperature of the top of the core was set to be 3800 K^7,35^, and the adiabatic geotherm of the mantle was taken from the literature^36^. We numerically calculated the thermal profile after thermal conduction over 10–200 million years (My) using a density of 5.5 g/cm^3 37^, a thermal conductivity of 10 W*m^− 1^*K^− 1 38^, and a specific heat capacity of 130 J*mol^− 1^*K^− 1 39^. We assumed that 200 My was a reasonable time scale according to plate motion, which was several centimetres per year and the depth of the CMB. We showed the maximum extent of the seismic wave velocity anomaly induced by the seifertite transition along the estimated slab temperature gradient (Fig. 3). The seismic wave velocities of the seifertite and CaCl_2_-type phases were taken from the literature^16^, and the effects of temperature and chemical factors, such as aluminium and hydrogen, were not considered here. This is a reasonable assumption because a recent study showed that the SiO_2_ phase contains a minor amount of aluminium in the dry subducted basalt^13^. The P-wave velocity increased by approximately + 1%, and the S-wave velocity decreased by approximately  -1% at the transition from CaCl_2_-type phase to seifertite. Conversely, at greater depths in the mantle, the P-wave velocity decreased by -1%, and the S-wave velocity increased by + 1% at the back transition from the seifertite to the CaCl_2_-type phase. The degree of anti-correlation in a natural slab is expected to be less significant than in the ideal case because the influences of thermal and chemical anomalies, as well as the post-perovskite phase transition, could also lead to seismic anomalies in addition to the seifertite transition. Nevertheless, the seifertite transition has a unique anti-correlation between the P- and S-wave velocities^16^, in contrast to the chemical heterogeneity, thermal heterogeneity, and the post-perovskite transition, which all show a positive correlation between the P- and S-waves^10,18^. If the magnitudes of the velocity anomalies differ substantially, the combination of multiple factors can coincidentally produce the anti-correlation. However, the magnitude of the anomaly is similar across plausible factors, including post-perovskite transition, temperature anomaly, and iron enrichment^8,18,40,41^, although the P-wave velocity has never been determined by Brillouin spectroscopy. The enrichment in SiO_2_ content, which is expected for the subducted slab, could be distinguished from other candidates of the anomalies by analysing both P- and S-wave velocity profiles. Notably, moreover, the post-perovskite phase transition does not show an abrupt anomaly because it occurs continuously over a wide pressure range in the MORB (Fig. S5)^9,42^. A recent theoretical study predicted that the anomaly in the P-wave at the seifertite transition can be a small negative value at higher pressure and temperature conditions, ranging from -2 to -4%, in contrast to the significant negative anomaly in the S-wave up to -11%^43^. The simultaneous determination of P-wave and S-wave is still challenging^17^, however, it is generally accepted that the S-wave velocity notably reduced at the seifertite transition^16,17,43^. Most recently, the Brillouin scattering study showed slower S-wave velocity for CaCl_2_-type and seifertite than the previous estimation^17^. Recent calculation studies suggest that the seifertite transition may decrease both P-wave and S-wave velocity, with a larger reduction in S-wave velocity, rather than producing an increase in P-wave velocity^43,44^. This indicates that the sign and magnitude of the P-wave velocity change remain uncertain and may depend on the computational approach, including the choice of exchange-correlation approximation. Further experimental and theoretical studies on the wave velocity of CaCl_2_-type and seifertite is necessary for a better understanding of the seismic anomaly. Since the MORB material could cause positive P- and S-wave anomaly by other candidates (such as post-perovskite transition) at CMB condition, the overall velocity structure should show anti-correlation in P- and S-wave, as a result of a significant negative anomaly in the P-wave at the seifertite transition. One may consider that the SiO_2_ component is limited in the subducting slab. However, it is suggested that the slab stagnates above 660 km depth to form a basalt-enriched “stagnant slab”, which eventually subducts into the lower mantle^1,45,46^ . Therefore, the subducting slab in the lower mantle may be enriched in SiO_2_, causing the anti-correlation.

### Seismic observations

**Fig. 4:**
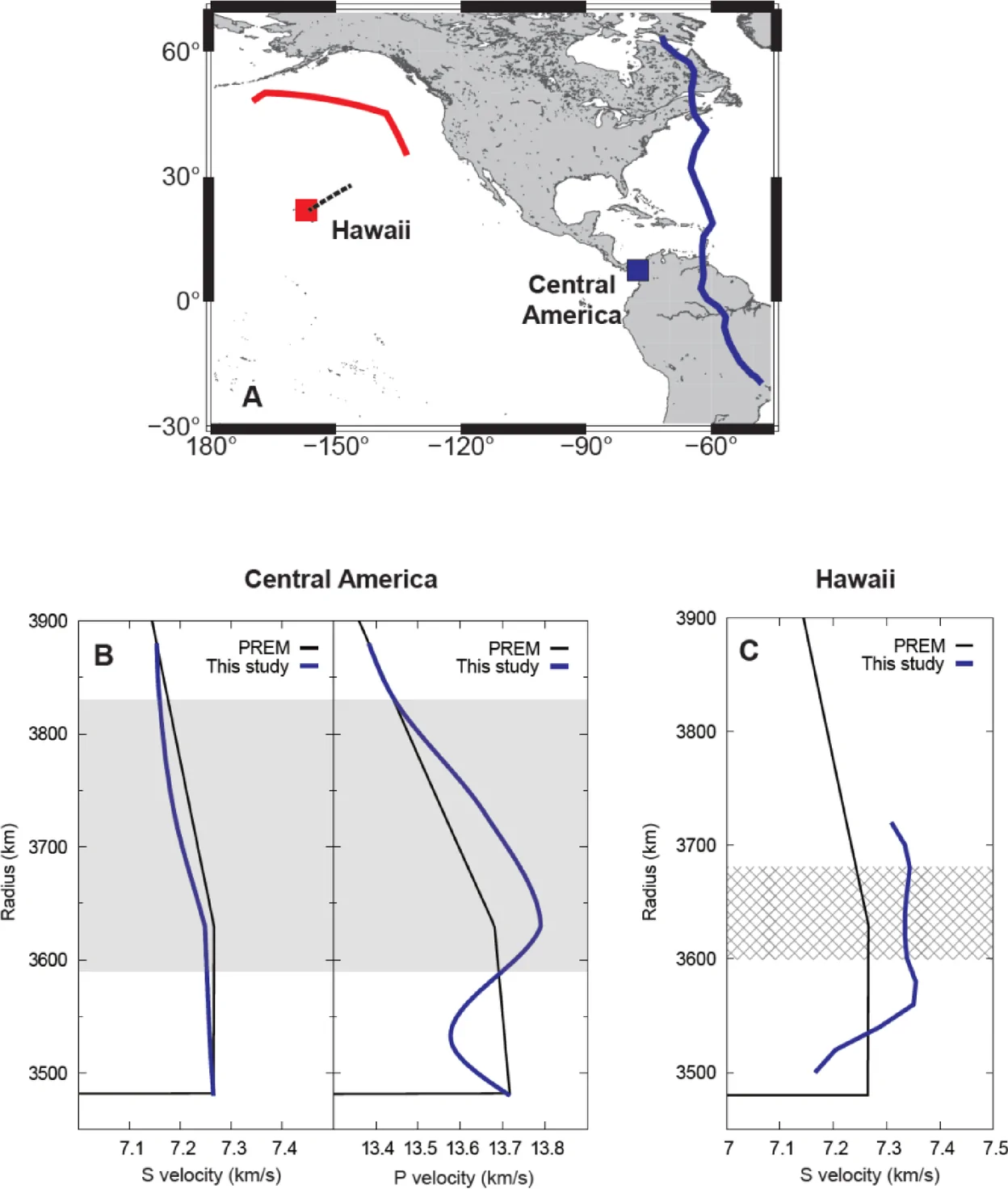
Seismic observations. (**A**) Locality of the seismic observation; blue (Central America) and red (Hawaii) squares show the latitude and longitude of the region of interest at the CMB depth, and the dashed line is the direction of the profile along the slab (see methods). Seismic wave velocity profile above the CMB beneath (**B**) Central America and (**C**) Hawaii. The thick blue lines show the S- and P-wave velocity profiles, and the black line shows the PREM. The grey bands are the depth range of seismic anomalies showing an anti-correlation caused by seifertite transition. The hatched area shows the depth range where S-wave velocity is slower relative to the above/below depth, possibly attributable to the seifertite transition.

To test the hypothesis, we compared the seismic wave velocity structures in the lowermost mantle beneath Hawaii and Central America inferred using the waveform inversion method for a large dataset of broadband seismograms (Fig. 4). Beneath Central America, a slow S-wave velocity anomaly was found 100–300 km above the CMB, clearly correlated with a fast P-wave anomaly. In contrast, both P and S waves exhibited slow anomalies at the lowermost mantle, extending from the bottom to 100 km above the CMB. These findings suggested that the CaCl_2_-type SiO_2_ phase transformed into seifertite 300 km above the CMB and reverted to the CaCl_2_-type phase 100 km above the CMB, because the chemical heterogeneity, thermal heterogeneity, and the post-perovskite transition can not cause the anti-correlation of P-wave and S-wave. On the other hand, beneath Hawaii, S-wave shows lower velocity at 100–200 km above the CMB, compared to the value above 200 km and below 100 km above the CMB. Although we have not obtained a P-wave velocity profile, the low S-wave anomaly along the slab beneath Hawaii was likely caused by the seifertite transition because (1) the post-perovskite phase transition induces a fast anomaly in the S wave^8,9^, and (2) the temperature anomaly in the observed narrow depth range (~ 100 km) is unlikely to be sustained for a geological time against heat conduction. The depth range of seifertite extended from approximately 100–300 and 100–200 km above the CMB beneath Central America and Hawaii, respectively. The temperature of the slab could reconcile these differences in the seifertite transition (Fig. 3). Although the observation beneath Hawaii is less supportive due to the absence of P-wave data compared to Central America, we conclude that the seismic anomalies beneath Central America and Hawaii are caused by the seifertite transition above the CMB in cold (Fig. 3A) and warm slabs (Fig. 3B), respectively. This finding may provide insight into the temperature of the slab around the CMB depth, yet this hypothesis should be further tested in future studies to better constrain the origin of the observed seismic anomaly.

Although this is outside our current scope, we observed that the S-wave velocity in Hawaii is considerably higher than that of Central America and PREM. This may be caused by large-scale chemical heterogeneity, such as a bridgmanite-rich pile on a > ~ 1000 km scale^47,48^. The current method is not suitable for detecting such large-scale chemical heterogeneity. However, we emphasise that we could obtain a wave-speed profile as a function of depth with high spatial resolution by applying waveform inversion to a large dataset of broadband seismograms, thereby providing possible evidence for SiO_2_-rich heterogeneity above the CMB.

## Discussion

### Implications for the deep mantle evolution

The seifertite double-crossing revealed that a cold slab descended towards the CMB beneath Central America (Figs. 3A and 4B). This observation aligns with the understanding that the western margins of North and South America are regions of prolonged subduction, where the oceanic plate is believed to have initiated eastwards subduction approximately 200 My ago^49^. The S-wave anomaly structure supports the idea that the plate has reached the CMB beneath Central America^50^. Conversely, the slab beneath Hawaii is expected to be warmer than that beneath Central America (Figs. 3B and 4C). This oceanic plate was subducted approximately 220 My ago from the intra-Panthalassa Ocean westwards subduction zone in the East Pacific, and it may have reached the CMB beneath Hawaii^2,3^. In contrast to Central America, the slab beneath Hawaii is likely to be relatively warm because of the high lateral heat flow from the nearby large low-shear-velocity province (LLSVP) located around the Hawaiian hot plume. Furthermore, the movements of slabs in the lower mantle may be more complex because of the presence of bridgmanite-enriched ancient mantle structures (BEAMS)^47^. The slab beneath Hawaii can be slow-moving because the westwards subduction conflicts with the upper mantle flow induced by BEAMS. In addition, the slab beneath Central America shows a steep dip angle, which could cause the colder subduction and rollback of the slab and its accumulation on the CMB, as suggested by mantle dynamics simulations^51^. The 500-K colder slab may have approximately 20–70% higher lattice thermal conductivity and approximately one order of magnitude higher viscosity than the warmer slab^52,53^. The highly viscous cold slab likely inhibited thermal convection in the mantle, leading to the formation of a slab accumulation region beneath Central America.

The SiO_2_ phase plays an additional important role in the dynamics of the CMB via the crystallisation of the oxide from the ancient core^54^. Core exsolved SiO_2_ may form a silica-rich body reaching 8.5 vol% of the mantle, causing chemical heterogeneity in the lower mantle^55^. Our results revealed that the CaCl_2_-type phase was stable under ancient hot CMB conditions, and subsequently, seifertite crystallised from the core as it cooled down. Dense seifertite is less buoyant than the CaCl_2_-type phase, and thus, the silica-rich body is gravitationally more stable and remains at the base of the mantle. The fate of the silica-rich body may have changed after the CMB temperature reached the seifertite stability field.

We address long-standing uncertainties in the seifertite phase transition and provide critical insights into slab dynamics at the CMB, and our results provide implications for understanding mantle convection and the thermal evolution of the Earth’s interior.

## Materials and methods

### High-pressure and high-temperature experiments

We prepared single-phase seifertite as a starting material using a multi-anvil press. Fine-powdered regent-grade SiO_2_ quartz was used to synthesise cristobalite at 1600 °C for 24 h using a cylindrical LaCrO_3_ heater. The sample was quenched by falling into the bottom of the furnace at room temperature. The recovered sample was a single-phase cristobalite. The synthesis of seifertite was performed at 22 GPa and 300 °C for 30 min in a 1000-ton Kawai-type multi-anvil press installed at the Institute for Planetary Materials, Okayama University. The sample pressure was determined using a pressure calibration curve at 1600 °C constructed with the akimotoite-bridgmanite transition^56^, wadsleyite-ringwoodite transition^57^, and forsterite-wadsleyite transition^58^. The cristobalite powder was put in a Pt-foil capsule. A Cr-doped MgO pressure medium with a 10-mm edge length was combined with tungsten carbide anvils with a 4-mm truncation to generate 22 GPa. A cylindrical LaCrO_3_ heater with two lids at both ends was used to generate high temperatures. A MgO capsule was located inside the heater. The sample capsule was put in the MgO capsule. A W-Re thermocouple (D-type) was measured at the surface of the sample capsule. The sample was first compressed to the pressure and then heated at a rate of 100 °C/min. After heating, the sample was quenched by switching off the electric supply and decompressed to ambient pressure for 12 h. The results of powder X-ray diffraction (XRD) measurements and a laser Raman spectrometer showed that the sample was composed of single-phase seifertite.

High-pressure and high-temperature experiments were carried out with laser-heated diamond anvil cells. Bevelled diamonds with 120–90 μm culet diameters were used for the anvils and rhenium was used for the gasket material. In the sample chamber, thin gold foil (0.8 μm thick) was sandwiched with pelleted seifertite. For the run conducted at approximately 133 GPa, a NaCl pressure medium was employed. In-situ XRD measurements were performed at SPring-8 BL10 XU^59^. Rapid (very short time interval and exposure time) XRD measurements were synchronised with a double-sided laser heating system and a spectrometer for temperature measurements. We achieved a 1000-fold increase in measurement speed compared with that of the conventional experimental setup^60^. The X-ray spot width was 6.5 μm in the vertical direction and 9.5 μm in the horizontal direction at the full width of the half-maximum. We used a LAMBDA 750k detector (X-Spectrum GmbH) for the XRD measurements. The temperature was measured via a spectroradiometric method with an HRS-300-SS spectrometer and a ProEM-HS:512B eXcelon EMCCD camera (Teledyne Princeton Instruments). The temperature readings were averaged over a width of 15 μm, which was approximately twice the X-ray spot size. Representative temperature profiles are shown in Fig. S6. We employed two fibre lasers (λ = 1064 nm, SP-100 C, SPI Lasers) integrated with an optical system that combined a focal π-shaper (Focal-πShaper 9_1964, Adl-Optica) and laser expanders to achieve a flat-top laser heating profile. A digital delay pulse generator (DG645, Stanford Research Systems) synchronised the laser heating and measurements. A function generator (EDU33210A, Keysight Technologies) was used to control the laser output. The heating duration ranged from 0.5 to 1 s, whereas the XRD measurements were conducted repeatedly with an exposure time of 0.02–0.2 s. The exposure times for the temperature measurements ranged from 0.01 to 0.2 s. In the run at around 133 GPa, additional prolonged heating was conducted for 5 min to ensure the stability of the seifertite. The obtained X-ray diffraction patterns were analysed by IPanalyzer, PDindexer, and Dioptas software^61,62^, and a custom Python script built with the pyFAI library^63^. The obtained unit cell parameters of the CaCl_2_-type phase and seifertite at high-pressures and -temperatures were consistent with those of previous studies^19,20,22^. After performing the high-pressure experiments, the recovered samples were further analysed by electron microscope. The cross sections of the samples were prepared using a focused ion beam attached to a scanning electron microscope (JIB4601F, JEOL). Elemental mapping was carried out by energy-dispersive X-ray spectroscopy (X-max 20mm^2^, Oxford Instruments), which revealed that the SiO_2_ sample retained its original chemical composition (Fig. S7).

We used the equation of state (EOS) for gold proposed by Fei et al.^64^, which is a widely accepted pressure scale based on the comprehensive range of data regarding pressure, temperature, and unit cell volume data of various calibrants. We obtained a slope of 13.3(9) MPa/K for the boundary. The uncertainty is estimated based on the alternative possible constraints using the third-closest experimental P-T conditions to the boundary. In addition, we compared the phase boundary with another pressure scale for gold proposed by Yokoo et al.^65^, which addresses extremely high pressure and temperature conditions through shock compression experiments. While we observed a slightly steeper phase boundary with Yokoo et al.’s pressure scale^65^, the slab temperature at the CMB remained considerably higher than temperatures that the seifertite boundaries locate (Fig. S3). Therefore, the choice of pressure marker did not fundamentally change our conclusions, although further research on the EOS under CMB conditions is warranted. Although the melting temperature of the gold is not yet clear above 100 GPa, our experimental temperature is generally consistent with the melting temperature of the gold^66^.

### Computational method

Density functional theory (DFT) calculations were conducted using the Q_UANTUM_ ESPRESSO code^67,68^. The exchange and correlation energies were described by the generalised gradient approximation (GGA) with the Perdew-Burke-Ernzerhof (PBE) scheme^69^. GBRV ultrasoft pseudopotentials^70^ were employed to reduce the computational costs by explicitly considering only the valence electrons in the configurations of 2*s*^2^ 2*p*^4^ and their cutoff radius (*r*_c_) of 1.25 a.u. for O and 3*s*^2^ 3*p*^2^ and *r*_c_ = 1.6 a.u. for Si. The kinetic energy cutoffs were determined to be 40 Ry (= 544.2 eV) for wavefunctions and 320 Ry (= 4353.6 eV) for charge density. The input supercells of seifertite and CaCl_2_-type SiO_2_ were orthorhombic, with cell parameters *a* = 4.097, *b* = 5.046, *c* = 4.946 Å for seifertite, and *a* = 3.8971, *b* = 4.0008, *c* = 2.5658 Å for the CaCl_2_ type SiO_2_. The Monkhorst-Pack k-point sampling scheme^71^ was employed with 4 × 4 × 4 grids for seifertite and 4 × 4 × 8 grids for CaCl_2_-type SiO_2_. The convergence thresholds of the total ion energy in the supercell and forces were determined to be 1.36 meV and 1.36 meV bohr^− 1^ ion^− 1^, respectively. To obtain the ground-state lattice energy *E*_lat_ for various volumes, variable-cell relaxations were conducted with the target pressures ranging from 0 to 150 GPa, with the convergence threshold for the stress being set to 0.05 GPa. In the variable-cell relaxations of the CaCl_2_ type, the structures obtained at a target pressure lower than 50 GPa showed a transition to stishovite; therefore, the target pressures for the CaCl_2_ type were limited at pressures higher than 50 GPa.

The Helmholtz free energy *F* of the system was approximated at an absolute temperature *T* as follows:

$$F\left( T \right){\text{ }} = E_{{{\mathrm{lat}}}} + F^{{ph}} \left( T \right),$$

where *F*^*ph*^ is the free-energy contribution of phonons^72^. In this system, the free-energy contribution of the electron was disregarded. Within the harmonic approximation, *F*^*ph*^ is expressed as $\:{F}^{ph}\left(T\right)={k}_{\mathrm{B}}T{\sum\:}_{s}\mathrm{ln}\left(2\mathrm{sinh}\frac{\hslash\:{\omega\:}_{s}}{2{k}_{\mathrm{B}T}}\right)$, where $\:{\omega\:}_{s}$ is a phonon frequency of vibration mode *s*^73^. Phonon calculations were conducted for these optimised structures using the density-functional perturbation theory (DFPT)^74^. Convergence of the free energy was checked by increasing the number of phonon wavevectors (*q* points). The calculated *F*(*T*) at several fixed volumes *V* was fitted to the isothermal Vinet equation of state (Vinet EoS)^75^, and the arbitrary pressure *P* was derived from the gradient as $\:P=-{\left(\frac{\mathrm{d}F}{\mathrm{d}V}\right)}_{T}$. The calculated Helmholtz free energies *F* of CaCl_2_-type SiO_2_ and seifertite are plotted in Fig. S1. The isothermal Vinet EoS was well fitted to *F* as a function of volume at temperatures ranging from 0 K to 4400 K. The volume at a fixed pressure decreased with increasing temperature. The Gibbs free energy of the system *G* was calculated as *G* (*P*, *T*) = *F* + *PV*. The calculated Gibbs free energies of CaCl_2_-type SiO_2_ and seifertite are compared in Fig. S2. The Gibbs free energy of seifertite was lower than that of CaCl_2_-type SiO_2_ at low temperatures and the boundary temperature increased with increasing pressure.

The free-energy calculations were used to determine the phase boundary by comparing the free-energy difference between phases; however, this approach cannot address the presence of metastable phases or crystal-size effects. If the thermal fluctuation of the potential energy exceeds the free-energy difference between CaCl_2_-type phase and seifertite, a higher-energy phase can crystallise as a metastable phase. Born-Oppenheimer molecular dynamics (BOMD) simulations were conducted under canonical (*NVT*) ensemble conditions via the velocity rescaling method. Following a simulation period of a few picoseconds, the temperature fluctuated around the target temperature without frequent velocity rescaling, and the conditions were nearly equal to those of the microcanonical (*NVE*) ensemble. The target temperature was set to 4000 K, and the volume was set to achieve a pressure of 135 GPa. The supercells included 108 SiO_2_, constructed from 3 × 3 × 3 of the primitive cell for seifertite and 3 × 3 × 6 for the CaCl_2_ type SiO_2_. The convergence thresholds of energy and forces were equivalent to those of the static calculations. The Γ point was utilised to sample the Brillouin zone. The equation of motion for the ions was solved by a differential equation through the Verlet algorithm with a time increment of 0.5 femtoseconds. The difference in the calculated Gibbs free energies between CaCl_2_-type SiO_2_ and seifertite was approximately 0.0003 Ry/SiO_2_ at 4000 K and 135 GPa near the core-mantle boundary conditions, as shown in Fig. S2. Although the stable phase was the CaCl_2_-type SiO_2_ phase, the difference in energy was very small. Therefore, the metastable phase could be locally crystallised and could lead to misleading results for the phase boundary. BOMD simulations could estimate whether the difference in energy was significant. The pressure and temperature of the CaCl_2_-type SiO_2_ and seifertite were set to 135 GPa and 4000 K, and the difference in pressure and temperature between these phases was negligible, as shown in Fig. S4. The average total energy *E*_tot_, which was the sum of the *E*_lat_ and kinetic energy, of CaCl_2_-type SiO_2_ was lower than that of seifertite (Fig. S4). This finding was consistent with the calculated difference in Gibbs free energy, as shown in Fig. S2. However, the fluctuation in energy with time was much larger than the energy variation range between these two phases. The fluctuation of the energy is mainly due to the fluctuation of the temperature. The standard deviation of temperature $\:\stackrel{-}{\varDelta\:T}$ satisfies the following relationship^76^:

1 $$\:\stackrel{-}{\varDelta\:T}=\sqrt{\frac{2}{3N}}\stackrel{-}{T}$$

where $\:\stackrel{-}{T}$ is the average temperature, *N* is the number of atoms. According to Eq. (1), the standard deviation $\:\stackrel{-}{\varDelta\:T}$ of 108 SiO_2_ (*N* = 324) at 4000 K can be estimated to be 180 K, which is consistent with that of the MD simulations ($\:\stackrel{-}{\varDelta\:T}$ = 150 K). This standard deviation $\:\stackrel{-}{\varDelta\:T}$ = 180 K corresponds to a kinetic energy of 0.005 Ry/SiO_2_, which is sufficiently larger than the difference in the Gibbs free energy between the seifertite and CaCl_2_-type phases under CMB conditions (0.0003 Ry/SiO_2_). Consequently, the metastable seifertite can be crystallised at 135 GPa and 4000 K within this small-scale number of atoms because thermal fluctuations easily overcome the minor difference in free energy. Utilising the Eq. (1) and the difference in the Gibbs free energy, it is deduced that the system of 30,000 SiO_2_ can overcome the difference in free energy to crystallise seifertite at this temperature and pressure. This size corresponds to approximately 8 × 8 × 8 nm^3^. In other words, intrinsic thermal fluctuations locally overcome the free energy difference to crystallise the metastable seifertite in the limited system of ~ 1000 nm^3^. This size was large enough to serve as a seed crystal for subsequent growth during the quenching process.

### Seismic observations

#### Central America

We previously obtained the 3-D S- and P-wave velocity structures in the lowermost mantle beneath Central America by applying the waveform inversion method to a large dataset of broadband seismograms recorded at the stations of the USArray^77^. We developed a new analytical method to infer the mantle P-wave velocity structure in the lowermost mantle with resolution comparable to that of the S-wave velocity structure. This was particularly challenging due to limited P-wave data sensitive to the area immediately above the CMB^77^. Our models revealed the following anomalous features beneath Columbia: (ⅰ) slow S- and fast P-wave velocity (anti-correlated) anomalies 300–100 km above the CMB and (ⅱ) slow S- and P-wave velocity anomalies 0–100 km above the CMB. We found the fast S- and P-wave velocity anomalies in the west-side region of the current locality^77^, which was interpreted as the Farallon slab subducted 180 My ago (Fig. 4)^49^. We interpreted the anti-correlated anomalies beneath Columbia as the SiO_2_-rich MORB supplied from the subducted slab. We show the vertical profiles of the S- and P-wave velocities beneath Columbia in Fig. 4.

#### Hawaii

In our previous study^78^, we applied the waveform inversion method to a large dataset of broadband seismograms to resolve the localised 3-D S-wave velocity structure in the lowermost mantle beneath the North Pacific. The obtained image revealed a sheet-like high-velocity zone ~ 150 km thick, supposedly a slab, reaching the CMB beneath Hawaii from the northeast. This slab was inferred to have subducted from an intra-oceanic subduction zone within Panthalassa (the paleo-Pacific) approximately 220 My ago (Fig. 4)^2,3^. We extracted S-wave velocity values from their model along the centre of this imaged slab, from a point 240 km above the CMB 10° northeast of Hawaii to a point just above the CMB directly beneath Hawaii (Fig. 4). The vertical profile of S-wave velocities along this slab is shown in Fig. 4.

## Supplementary Information

Supplementary Information 1.

Supplementary Information 2.

## Data Availability

XRD raw spectra data are available in the supplementary information files. The datasets used and/or analysed during the current study are available from the corresponding author on reasonable request.
