# Supplementary material for: Cold SiO2-rich slabs reaching the CMB revealed by the seifertite phase boundary
Source: Sci Rep. 2026 May 30;16:26394. doi: 10.1038/s41598-026-54731-6 (PMC13500678; doi:10.1038/s41598-026-54731-6)
Supplement: Supplementary file 1 [file 41598_2026_54731_MOESM2_ESM.docx]

Supplementary Materials for

**Cold SiO_2_-rich slabs reaching the CMB revealed by the seifertite phase boundary**

Ryosuke Sinmyo* *et al.*

*Corresponding author. Email: [sinmyo@meiji.ac.jp](mailto:sinmyo@meiji.ac.jp)

**Fig. S1. Helmholtz free energy of SiO_2_ phases.**

Helmholtz free energy of (**A**) CaCl_2_-type SiO_2_ and (**B**) seifertite as a function of volume and temperature. The symbols indicate the calculated free energy at different volumes and temperatures from 0 K to 4400 K every 200 K. The solid lines are the results of fitted Vinet EoSs. The dashed lines indicate the isobaric points calculated from the gradient of the Vinet EoSs.

Fig. S2. Gibbs free energies of SiO_2_ phases.

Gibbs free energies of CaCl_2_-type SiO_2_ (*G* (CaCl_2_)) and seifertite (*G* (Sei)), and the differences at pressures of 100, 110, 120, 130, 135, and 140 GPa. The green area indicates the temperatures at which seifertite is more stable than CaCl_2_-type SiO_2_.

Fig. S3. Comparison of the seifertite boundary with previous studies.

CaCl_2_-type phase to seifertite phase boundaries of this study and previous studies. Red lines, this study; blue lines, previous experimental studies; green lines, previous theoretical studies. The grey region shows the pressure and temperature at which the two phases of the CaCl_2_-type phase and seifertite were observed in previous experimental studies (*19, 22*). The red solid and dotted lines represent our results when the Au pressure marker of Fei et al. (*58*) and Yokoo et al. (*59*), were used, respectively. The dashed red line represents the theoretical calculations in this study. The thin dotted line represents the pressure condition of the CMB.

**Fig. S4. Energetic difference between SiO_2_ phases at CMB conditions.**

Time development of pressure (top), temperature (middle), and total energy (bottom) in BOMD simulations of CaCl_2_-type SiO_2_ and seifertite. The solid circles represent the average values of CaCl_2_-type SiO_2_ (red) and seifertite (black), with their respective standard deviations.

Fig. S5. Comparison of the seifertite boundary with post-perovskite boundary.

Comparison of the phase boundaries between post-perovskite and seifertite. The red dashed line is the seifertite boundary in this study. The solid black line is the boundary in MgSiO_3_ _(_*_33_*_)_, and the grey regions are regions where bridgmanite and post-perovskite coexist in the MORB (O08 (*9*) and G12 (*40*)). The thick grey broken line is a plausible temperature profile of the cold slab (see text).

Fig. S6. Temperature profile of the diamond anvil cell experiments.

Temperature profile across laser heating spot in the laser-heated diamond anvil cell experiments. The filled and open symbols represent the upside and downside of the diamond anvil cell along the X-ray pass, respectively. Broken lines show the width of the X-ray in both the horizontal (9.5 μm) and vertical directions (6.5 μm) (*53*).

**Fig. S7. Scanning electron microscopy image.**

Scanning electron microscopy image of the recovered sample from 162 GPa and 5400 K. (**A**) Wide view of the sample with a flat surface polished by the focused ion beam. The broken line shows the outline of the sample, and the chain line shows the region of the X-ray mapping analysis. The downside of the sample was mostly lost during the decompression. (**B**) High-resolution electron microscopic image with the sample outlines (broken lines). X-ray maps of (**C**) Si, (**D**) O, and (**E**) Au obtained via EDS analysis.

| **Table S1. Experimental conditions and results.** | | |  |  |
| --- | --- | --- | --- | --- |
| Pressure (GPa) | Temperature (K) | Measurement time (seconds) | | Stable phase |
|  |  | XRD | Specftroradiometry |  |
| 121(4) | 3590(461) | 0.20 | 0.20 | CaCl_2_-type SiO_2_ |
| 122(2) | 3840(212) | 0.20 | 0.20 | CaCl_2_-type SiO_2_ |
| 133(3) | 3200(331) | 0.20 | 0.20 | seifertite |
| 134(7) | 5340(848) | 0.10 | 0.05 | CaCl_2_-type SiO_2_ |
| 153.2(2) | 4834(23) | 0.02 | 0.01 | CaCl_2_-type SiO_2_ |
| 155(1) | 5110(101) | 0.02 | 0.01 | CaCl_2_-type SiO_2_ |
| 162(3) | 5360(408) | 0.01 | 0.01 | seifertite |
| 164(2) | 5880(276) | 0.10 | 0.05 | CaCl_2_-type SiO_2_ |
| 178(1) | 6010(157) | 0.02 | 0.01 | seifertite |

Table S1. Experimental conditions and results.
